# A simple and accurate PCR method for detection of genetically modified rice

**DOI:** 10.1007/s40201-019-00401-x

**Published:** 2019-10-31

**Authors:** Payam Safaei, Ebrahim Molaee Aghaee, Gholamreza Jahed Khaniki, Setareh Agha Kuchak Afshari, Sassan Rezaie

**Affiliations:** 1 Department of Environmental Health Engineering, School of Public Health, Tehran University of Medical Sciences, Tehran, Iran; 2 Student’s Scientific Research Center, Tehran University of Medical Sciences, Tehran, Iran; 3 Department of Medical Parasitology and Mycology, School of Medicine, Kerman University of Medical Sciences, Kerman, Iran; 4 Department of Medical Mycology and Parasitology, School of Public Health, Tehran University of Medical Sciences, Tehran, Iran

**Keywords:** Genetically modified rice, Detection method, PCR

## Abstract

**Background:**

Legislation regulating for labeling and use of genetically modified (GM) crops are increased considerably worldwide in order to health and safety assurance of consumers. For this purpose, a polymerase chain reaction (PCR) method has been developed for detection of GM rice in people’s food diet.

**Methods:**

In this study, eighty-one non-labeled rice samples were collected randomly from different market sites of Tehran, Iran. In order to analysis, rice genomic DNA was extracted using MBST DNA extraction kit and subsequently, sucrose phosphate synthase (SPS) gene was used to confirm the quality of extracted DNA. Then, cauliflower mosaic virus (CaMV) 35S promoter and Agrobacterium nopaline synthase (NOS) terminator were selected as screening targets for detection of GM rice sequences by PCR.

**Results:**

According to our results, 2 out of 81 (2.4%) samples tested were positive for CaMV 35S promoter while no positive result was detected for NOS terminator.

**Conclusion:**

The obtained data indicated that this method is capable to identify the GM rice varieties. Furthermore, it can demonstrate the possibility of the presence of GM rice in Tehran’s market, thus putting emphasis on the requirement for developing a precise approach to evaluate this product.

## Background

Rice is one of the major crops cultivated in the world. Almost 50% of the world’s population depend on rice for their body calories [1]. Compared to traditional method, genetic engineering can be used to rise and stabilize yield, herbicide tolerance, disease and insect resistance, nutritional improvements and withstanding to abiotic stresses. The genetically modified (GM) rice was developed for the first time by plant transformation methods in 1988 [2, 3]. The aims of GM rice production were improvement of quality and reduction in pesticides or herbicides used in the fields, that could not be achieved through other breeding methods [4, 5]. The global area cultivated with GM crops was 185.1 million hectares in 2016. In spite of most engineering plants (including soybean, maize, cotton, and canola) there are only few number authorized transgenic rice worldwide, that more developed in Asia, although these GM varieties are not approved for commercialization. Despite regulations, unauthorized GM rice has been detected in many countries [6]. China is the largest rice producer country in the world, and 20% of planted area is devoted to rice cultivation [7]. In Iran, a transgenic rice that has been genetically modified by introducing Cry1Ab gene from the bacterium *Bacillus thuringiensis*, commercially cultivated 13 years ago but is currently not authorized to cultivation. This gene increase the plant’s resistant to insects and lead to growing production [8].

Along the benefits of GM Crops, biodiversity, increas of insect resistance, herbicide tolerance and human health risk are the most potential concerns of these food materials [9]. In addition, in general the health risk assessment of inserted gene into food materials for humans has not been systematically shown in the literature. Thus, their detection and labelling is required for increasing the consumer awareness [10]. Therefore, due to growing the number of unauthorized GM rice varieties in the market and ethical issues about providing informed choice to the consumer, development of screening methods and monitoring programs seems to be essential in this scope. DNA-based PCR is the accurate and most widely used method for GMO testing [11–13]. Moreover, in compared to other methods such as the enzyme-linked immunosorbent assay (ELISA), PCR has higher specificity to acquire reliable results [14].

*Cauliflower mosaic virus* (*CaMV*) 35S promoter (P-35S) and nopaline synthase terminator (T-nos) from *Agrobacterium tumefaciens* are the most common transgenic elements that can be targeted for GMO screening [2, 14–16]. These genetic elements are frequently used to indicate whether the analyzed sample contains GM ingredient or not. In addition, the proper Certified Reference Material (CRM) is necessary as a positive control for validation of GMO detection method [17, 18].

The number of studies based on PCR methods has been applied to GMO detection. For example, in Brazil, food samples were analyzed for GMO screening. Those results showed that some of the food crops tested have been genetically modified [19]. In other work, Arun et al. (2013) found CaMV 35S promoter, and the NOS terminator in 25% of the collected products [20]. In Iran, it is estimated that about 2.2 million tons of rice are cultivated in 2017. Moreover, Iran has imported over 1million tons of rice during this year [21]. To our knowledge, the control of this crop mainly depends on the heavy metal pollution, although according to regulatory authorities, it is forbidden both to cultivate or import GM rice. The aim of present study was to determine an acceptable and cost-effective PCR assay for detection of transgenic rice in Tehran market.

## Materials and methods

### Sample preparation

Two certified reference materials (CRMs) were obtained from the Institute of Reference Materials and Measurements (IRMM, Geel, Belgium) in the EU. These references were two available GM varieties (Bt 11 maize 5% and Roundup Ready soy 5%), which GM target sequences are present in both, thus have been used as the positive controls in the present study. Eighty-one rice seed samples (each sample 500 g) were purchased randomly from various local markets in Tehran, during 2018. All the samples were homogenized using an electric homogenizer and stored at −20°c before DNA extraction.

### DNA extraction and qualification

Seed samples and references were grounded with an electric grinder. Genomic DNA was extracted from all samples using the DNA Extraction kit from Plant Materials (MBST, IRAN) according to the instruction, some adjustments were also used to improve the quality of DNA. Briefly, one hundred milligrams of the powder transfer into the clean Eppendorf tube containing 300 μl lysis buffer. 20 μl proteinase K was added into the mixture and incubated for 15 min at 60 °c. After incubation 580 μl binding buffer was added to the tube, mixed by vortexing and incubated for 10 min at 70 °c, then centrifuged for 1 min by 8000×*g* and transferred the supernatant into the clean 1.5 ml Eppendorf tube. Placed a spin column (A) in a 1.5 ml Eppendorf tube and then supernatant applied to the spin column (A) and centrifuged at 8000×*g* for 1 min, removed the column (A), then 440 μl ethanol (100%) added into the solution and Followed by applied into the new spin column B and centrifuged at 8000×*g* for 1 min. After centrifugation, 500 μl wash buffer added to column B and centrifuged at 8000×*g* for 3 min to remove the ethanol completely. After the wash, the tube containing infiltrate discarded. Placed the spin column B in a clean Eppendorf tube and added 35–50 μl elution buffer preheated to 70 °c to the column, incubated at room temperature for 3 min, then centrifuged at 8000×*g* for 1 min. The solution obtained, stored at −20°c prior to screening.

The purity and quality of isolated DNA is the main step to the efficiency of the PCR [22]. The concentration and purity of extracted DNA was evaluated by ultraviolet (UV) absorption at wavelengths of 260 nm and 260/280 nm using a NanoDrop spectrophotometer, respectively.

### Oligonucleotide primers

Four primer pairs have been used for detection of GM rices. Moreover, the sucrose phosphate synthase (SPS), soy Lectin and maize invertase genes were used as rice-specific, soy-specific, and maize-specific endogenous reference genes, respectively. Therefore, the tree primer pairs including SPS-F/R, Lectin-F/R and Invertase-F/R were used for verification of the presence and quality of extracted DNA from rice samples, soy CRM, and maize CRM, respectively. The DNA quality determination of CRMs is required to detect the GM rice samples accurately. Since these materials are presented as a control group during the study. The primer pairs P-35S and T-nos were prepared according to the international standard organization (ISO 21569: 2005) guideline [23]. The sequences of the SPS, Lectin and Invertase genes for primer designing were obtained based on previously published papers [19, 24]. The target sequences were also analyzed using NCBI primer-BLAST search to verify the specificity of the primers. The information of primers used is shown in Table 1.

**Table 1:** Primer pairs used in this study

**Primer name**	**Sequence (5′ – 3′)**	**target**	**Length (bp)**	**Reference**
SPS-F	TTG CGC CTG AAC GGA TAT	SPS	277	[23]
SPS-R	GGA GAA GCA CTG GAC GAG G			
P35S-cf3	CCA CGT CTT CAA AGC AAG TGG	P-35S	123	[19, 24]
P35S-cr4	TCC TCT CCA AAT GAA ATG AAC TTC C			
HA-nos-118f	GCA TGA CGT TAT TTA TGA GAT GGG	T-NOS	118	[19, 24]
HA-nos-118r	GAC ACC GCG CGC GAT AAT TTA TCC			
GM03	GCC CTC TAC TCC ACC CCC ATC C	Lectin	118	[23]
GM04	GCC CAT CTG CAA GCC TTT TTG TG			
IVR1-F	CCG CTG TAT CAC AAG GGC TGG TAC C	invertase	226	[23]
IVR1-R	GGA GCC CGT GTA GAG CAT GAC GAT C			

### PCR conditions

The PCR analysis were carried out in a thermal cycler (96 universals, PEQStar, Germany). Amplification reactions contained 2 μl of genomic DNA and appropriate PCR reaction mixture. PCR reaction mixture including: 12 μl ready-to use PCR master mix 2× (The composition: Tris-Hcl pH 8.5, (NH4)2SO4, 3 mM Mgcl2, 0.2% Tween 20, 0.4 mM dNTPs, 0.2 units/μl Ampliqon Taq DNA polymerase, Inert red dye and stabilizer), 1 μl of each primer, and 9 μl sterile free ions distill water. The concentration of primers for all target gene was 0.1 μl. Finally, PCR assays were performed in a volume of 25 μl. The reaction conditions of PCR were as follows: For SPS: initial denaturation at 94 °C for 5 min, amplification at 94 °C for 30 s, annealing at 58 °C for 45 s, extension at 72 °C for 75 s, and a final elongation for 8 min at 72 °C. For GM03/GM04: initial denaturation at 94 °C for 5 min, amplification at 94 °C for 1 min, annealing at 60 °C for 40 s, and a final elongation for 8 min at 72 °C. For IVR1-F/IVR1-R: initial denaturation at 94 °C for 5 min, amplification at 94 °C for 1 min, annealing at 64 °C for 40 s, and a final elongation for 8 min at 72 °C. For P35S-cf3/P35S-cf4 and HA-nos-118f/ HA-nos-118r: initial denaturation at 94 °C for 5 min, amplification at 94 °C for 1 min, annealing at 60 °C for 40 s, and a final elongation for 8 min at 72 °C.

### Agarose gel electrophoresis

Eight μl of PCR products (including PCR amplification products, positive and negative controls) were electrophoresed on 2% agarose gel containing 2 μl DNA safe stain at 80 Voltage in 1× TBE running buffer (containing 600 ml dH2O, 48.4 g Tris base, 11.42 ml glacial acetic acid and 40 ml EDTA (0.5 M), PH 8.0, then dilute with dH2O to obtain a final volume of 1 L) for 60 min. 4 μl of 100 bp DNA ladder was used as a reference marker. DNA fragments were separated through a gel based on size and then visualized using UV- transluminator.

## Results and discussion

**Fig. 1:**
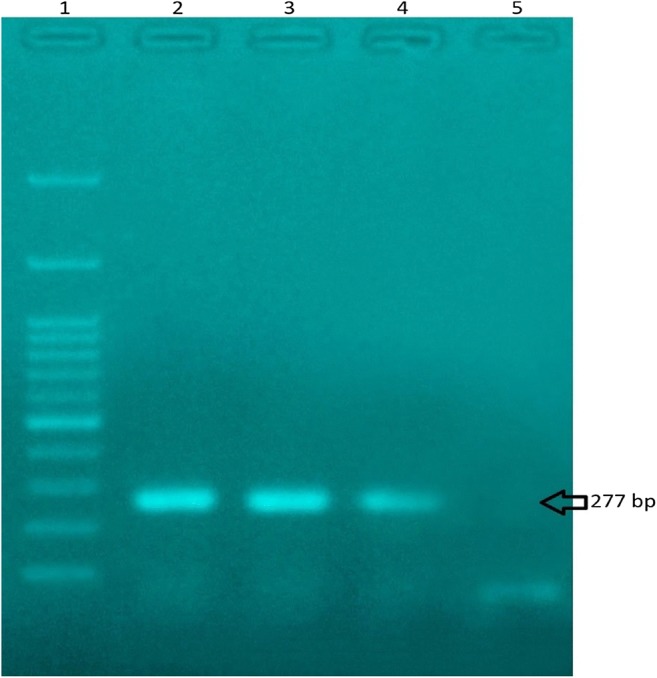
Agarose gel electrophoresis of PCR products from rice samples; 1: 100 bp DNA ladder, 2: Standard rice sample, 3: Rice sample 1, 4: Rice sample 2, 5: sterile water

In present study, about 40 μl DNA solution was obtained from rice seeds and reference materials using DNA extraction kit (MBST, IRAN). The range of absorbance ratio to quality control and concentration of DNA extracted for both sample types was between 1.7 and 1.9. But in some cases, for achieving to this range we repeated attempts. Based on previous studies, this amount is acceptable for PCR amplification [25, 26]. On the other hand, as an internal positive control, the SPS endogenous PCR products was amplified to confirm the presence of a sufficient amount of DNA from Rice samples (Fig. 1). Moreover, to reduce false positive results during the extraction of DNA or PCR analysis, sterile water was used in parallel to the sample preparation and analysis.

**Fig. 2:**
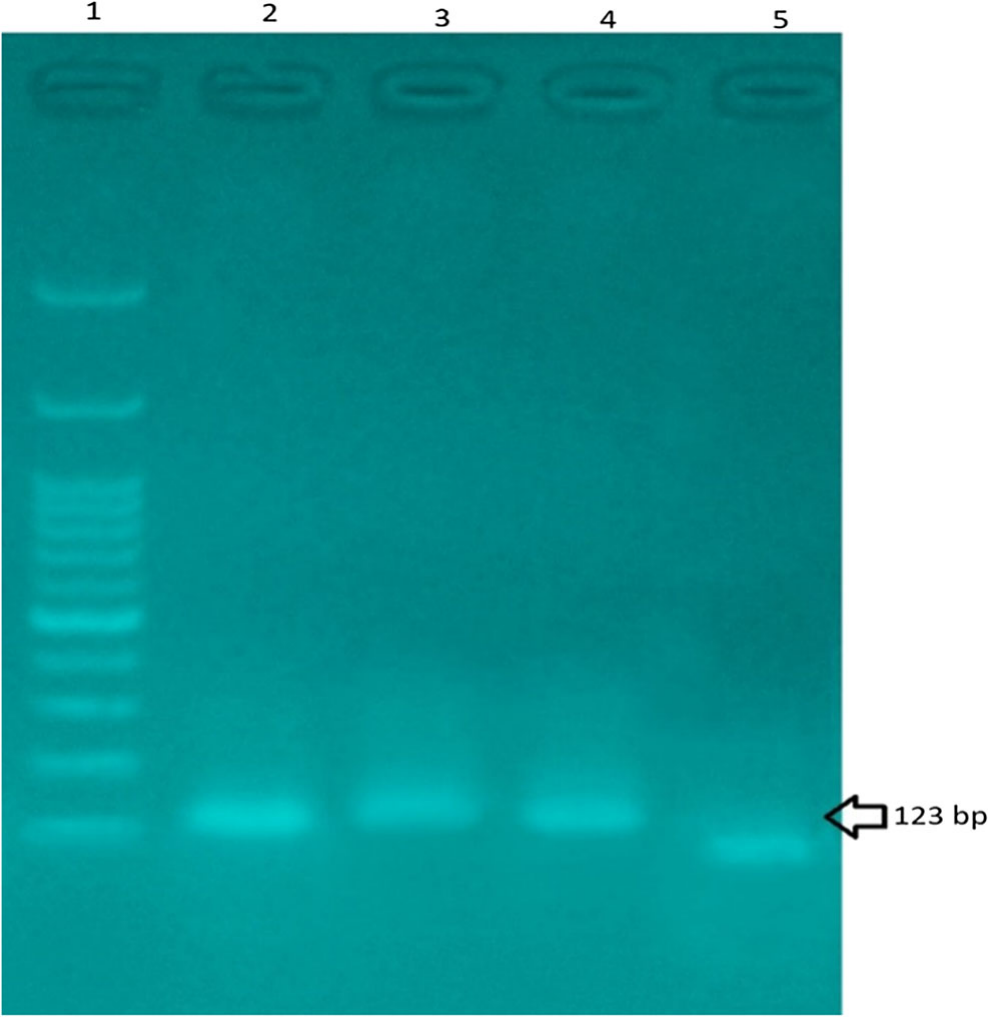
Agarose gel electrophoresis of PCR products from rice samples; 1: 100 bp DNA ladder, 2: positive control, 3: Rice sample 1, 4: Rice sample 2, 5: Negative control

All of the 81-positive sample Rice screened for SPS gene were analyzed for detection of the CaMV 35S promoter and NOS terminator which indicates the presence of GMO targets. The control of these samples was performed with extracted DNA from GM soy. The CaMV 35S promoter sequences was observed only in two DNA rice samples in a size of 123 bp using a primer pair P35S-cf3/P35S-cf4. While none of the analyzed samples were positive for the presence of NOS terminator using primer pair HA-nos-118f/ HA-nos-118r at the size of 118 bp. Similarly, other researchers declared the possibility of GMO detection by PCR method and these primer pairs [27, 28]. The results of gel electrophoresis for positive samples are shown in Fig. 2. To ensure the results are achieved, nucleotide sequences of the PCR products with DNA extracted of positive samples were determined. The results of sequencing, were analyzed with BLAST search of the NCBI and revealed that 2 of the 81 samples contained 35S promoter. In the other research, Arun et al. (2013) screened maize and soy in processed foods for detection of CaMV 35S promoter and nos terminator by PCR technique, the results indicated that 25 of the 100 (25%) samples were GM positive [20]. The Oraby et al. (2005) reported that 12.5% of the food product tested gave the positive result to CaMV 35S, while negative results were determined for NOS primer [29]. Similarly, Erkan and Dastan (2017) found that 11 samples of the rice and rice flour products contained GM targets [30]. However, in order to determine the transgenic rice event, further event-specific method is required. On the other side, because of safety issues, rice events are not approved in Iran and most of countries, consequently their consumption may have undesired effect on human health. Xue et al. (2013) in a risk assessment study have stated that biotech rice might increase the concerns about risks related to the human health [31]. A study conducted in china showed that 1 out of two rice samples tested was positive for CaMV 35S promoter [11]. In another study, two hundred samples (including maize, soy, and rice) evaluated for detection of genetically modified. Two primer pair p35S and NOS were used to detection strategy. Those result indicated that 26 and 44% of samples containing soybean and maize were positive respectively, in contrast, all of the rice samples were negative for these two primers [9]. Other researchers also concluded that successful screening of GMO targets in food products by PCR is achievable and preferred than other methods [32, 33]. In our study, 2 out of 81 rice samples showed positive results for primer pair P35S-cf3/P35S-cr4, therefore it may to be genetically modified, while none of them has been labeled. However, because of the assessment of Food and Drug Administration, the positive results might be due to illegal entry of this product to Iran or their infected by the CaMV virus.

## Conclusion

The result of this study demonstrated that conventional PCR can be an appropriate method for screening of GMO targets. Also, we can conclude that DNA isolation method and primer designed was satisfactory for sample analysis. Based on our results few sample rices were genetically modified. However, lack of the available CRM of GM rice is the limitation of this study. Due to consumer’s concern regarding the safety of genetically modified crops, labeling is required in order to make informed decisions. For the reasons stated above, establishing of the regulation and monitoring system in Iran is recommended.
